# Supplementary material for: Nanosurface Texturing for Enhancing the Antibacterial Effect of Biodegradable Metal Zinc: Surface Modifications
Source: Nanomaterials (Basel). 2023 Jul 7;13(13):2022. doi: 10.3390/nano13132022 (PMC10343544; doi:10.3390/nano13132022)
Supplement: Supplementary file 1 [file nanomaterials-13-02022-s001.zip › nanomaterials-2449666-supplementary.pdf]

nanomaterials

Supplementary Materials

Nanosurface Texturing for Enhancing the Antibacterial Effect of Biodegradable Metal Zinc: Surface Modifications

Enmao Xiang [1,2], Corey S. Moran [1,2], Sašo Ivanovski [1,2,*] and Abdalla Abdal-hay [1,2,3,4,*]

[1] School of Dentistry, The University of Queensland, Brisbane 4006, Australia; uqexiang@uq.edu.au (E.X.); corey.moran@uq.edu.au (C.S.M.)
[2] Centre for Orofacial Regeneration, Reconstruction and Rehabilitation (COR3), School of Dentistry, The University of Queensland, Brisbane 4006, Australia
[3] Department of Engineering Materials and Mechanical Design, Faculty of Engineering, South Valley University, Qena 85325, Egypt
[4] Faculty of Industry and Energy Technology, Mechatronics Technology Program, New Cairo Technological University, Fifth Settlement, Cairo 11835, Egypt
* Correspondence: s.ivanovski@uq.edu.au (S.I.); abdalla.ali@uq.edu.au (A.A.-h.)

Table S1. Peak analysis data for XRD of pure untreated, 5% etched, and 15 etched Zn.

	Pure Zn	5% etched Zn	15% etched Zn	Pure Zn	5% etched Zn	15% etched Zn	Pure Zn	5% etched Zn	15% etched Zn	Pure Zn	5% etched Zn	15% etched Zn	Pure Zn	5% etched Zn	15% etched Zn
2θ (°)		d (Å)			I			h			K			I	
36.37	2.47	2.47	2.47	392	392	391	0	0	0	0	0	0	2	2	2
39.08	2.30	2.30	2.31	236	235	234	1	1	1	0	1	0	0	0	0
43.32	2.09	2.09	2.09	1000	1000	1000	1	1	1	0	1	0	1	1	1
54.44	1.68	1.68	1.69	146	146	146	1	1	1	0	1	0	2	2	2
70.24	1.34	1.34	1.34	157	156	155	1	1	1	0	0	0	3	3	3

70.80	1.33	1.33	1.33	102	101	101	1	1	1	1	1	1	0	0	0
77.23	1.23	1.23	1.24	24	24	24	0	0	0	0	1	0	4	4	4
82.29	1.17	1.17	1.17	110	109	110	1	1	1	1	0	1	2	2	2
83.97	1.15	1.15	1.15	13	13	13	2	2	2	0	0	0	0	0	0
86.76	1.12	1.12	1.12	67	66	66	2	2	2	0	0	0	1	1	1
90.16	1.09	1.09	1.09	19	19	19	1	1	1	0	0	0	4	4	4
95.14	1.04	1.04	1.04	16	16	16	2	2	2	0	0	0	2	2	2
